# Supplementary material for: Mortality from sickle cell disease in Brazil
Source: PLOS Glob Public Health. 2025 Jul 24;5(7):e0002066. doi: 10.1371/journal.pgph.0002066 (PMC12289050; doi:10.1371/journal.pgph.0002066)
Supplement: S1 Table — (DOCX) [file pgph.0002066.s001.docx]

| **Adults who died according to iron overload & chronic transfusion therapy status** | | | |
| --- | --- | --- | --- |
| **Chronic transfusion therapy** | **Iron overload** | **No iron overload** | **Total** |
| **Yes** | 18 (13.8%) | 1(0.8%) | 19 (14.6%) |
| **No** | 23(17.7%) | 88(67.7%) | 111 (85.4%) |
| **Total** | 41 (31.5%) | 89 (68.6%) | 130 (100) |
